# Regulation of cisplatin-resistant head and neck squamous cell carcinoma by the SRC/ETS-1 signaling pathway

**DOI:** 10.1186/s12885-019-5664-7

**Published:** 2019-05-22

**Authors:** Zejia Yang, Jipei Liao, Brandon A. Carter-Cooper, Rena G. Lapidus, Kevin J. Cullen, Hancai Dan

**Affiliations:** 1 Marlene and Stewart Greenebaum Comprehensive Cancer Center, University of Maryland School of Medicine, Baltimore, MD USA; 2 Department of Pathology, University of Maryland School of Medicine, Baltimore, MD USA

**Keywords:** Head and neck squamous cell carcinoma, HNSCC, ETS-1, Cisplatin resistance, SRC, MEK/ERK, Dasatinib

## Abstract

**Background:**

We investigated the role of the ETS-1 transcription factor in Head and Neck Squamous Cell Carcinoma (HNSCC) in multiple cisplatin-resistant HNSCC cell lines.

**Methods:**

We examined its molecular link with SRC and MEK/ERK pathways and determined the efficacy of either MEK/ERK inhibitor PD0325901 or SRC inhibitor Dasatinib on cisplatin-resistant HNSCC inhibition.

**Results:**

We found that ETS-1 protein expression levels in a majority of cisplatin-resistant HNSCC cell types were higher than those in their parental cisplatin sensitive partners. High ETS-1 expression was also found in patient-derived, cisplatin-resistant HNSCC cells. While ETS-1 knockdown inhibited cell proliferation, migration, and invasion, it could still re-sensitize cells to cisplatin treatment. Interestingly, previous studies have shown that MER/ERK pathways could regulate ETS-1 through its phosphorylation at threonine 38 (T38). Although almost all cisplatin-resistant HNSCC cells we tested showed higher ETS-1 phosphorylation levels at T38, we found that inhibition of MEK/ERK pathways with the MEK inhibitor PD0325901 did not block this phosphorylation. In addition, treatment of cisplatin-resistant HNSCC cells with the MEK inhibitor completely blocked ERK phosphorylation but did not re-sensitize cells to cisplatin treatment. Furthermore, we found that, consistent with ETS-1 increase, SRC phosphorylation dramatically increased in cisplatin-resistant HNSCC, and treatment of cells with the SRC inhibitor, Dasatinib, blocked SRC phosphorylation and decreased ETS-1 expression. Importantly, we showed that Dasatinib, as a single agent, significantly suppressed cell proliferation, migration, and invasion, in addition to survival.

**Conclusions:**

Our results demonstrate that the SRC/ETS-1 pathway plays a crucial role and could be a key therapeutic target in cisplatin-resistant HNSCC treatment.

**Electronic supplementary material:**

The online version of this article (10.1186/s12885-019-5664-7) contains supplementary material, which is available to authorized users.

## Background

Head and neck squamous cell carcinoma (HNSCC) ranks as the sixth most common cancer in the world, with a 5-year survival rate of less than 50%. The high mortality mainly occurs because of poor prognosis in patients with recurrent or/and metastatic HNSCC. Cisplatin-containing chemotherapy is the first option to treat recurrent and metastatic HNSCC. Unfortunately, this treatment only achieves a good response for a short time because a majority of patients develop resistance to cisplatin and will die within one year [1–4]. Therefore, in order to identify the crucial oncogenic and survival proteins and signaling pathways associated with HNSCC recurrence and metastasis, development of agents to counteract cisplatin resistance remains a major focus in HNSCC research.

The ETS-1 transcription factor is a 54 kDa nuclear protein that functions as a transcriptional activator. It plays a role in cancer progression through a number of processes, which include regulation of proliferation, invasion, epithelial-to-mesenchymal transition (EMT), metabolism, angiogenesis, and drug resistance in many types of cancers [5]. It has been reported that ETS-1 plays an important role in promoting tumor invasion in both laryngeal [6] and oral squamous cell carcinomas [7, 8]. ETS-1 expression is also involved in metastasis of undifferentiated nasopharyngeal carcinomas [9]. Although ETS-1 may play a role in chemotherapy resistance in lung, ovarian, and colorectal cancers, its precise role in cisplatin-resistant HNSCC has not been well documented [5].

In addition, identification of crucial regulators upstream of ETS-1 is also important to effectively inhibit its function in cisplatin-resistant HNSCC. An increasing number of studies demonstrated that ETS-1 stability and transcription activity were regulated by phosphorylation of multiple protein kinases in lung and breast cancers [5]. The most common protein kinases linked to ETS-1 include the MAPK/ERK (MEK/ERK) and SRC kinases [5]. Intensive studies have shown that the MEK/ERK signaling pathway could regulate ETS-1 through phosphorylation at threonine 38 in lung and breast cancers [5, 10–14]. Recently, it was also shown that SRC phosphorylation of ETS-1 at tyrosine 283 released COP1 degradation of ETS-1 in breast cancer [5, 15]. To date, however, the precise roles of the MEK/ERK and SRC pathways through ETS-1 regulation remain unknown in cisplatin-resistant HNSCC.

In this study, we determined the role of ETS-1 in multiple cisplatin-resistant HNSCC. Our data indicate that ETS-1 plays important roles in the regulation of cell proliferation, survival, migration, and invasion downstream of SRC in a MEK/ERK-independent manner.

## Methods

### Cell culture

HNSCC cell lines Cal 27, FaDu, and SCC25 were obtained from ATCC. UMSCC17B, UMSCC2, and UMSCC74B cell lines were the generous gift of Dr. Thomas E. Carey (University of Michigan, Ann Arbor, MI, USA). These cell lines (Cal 27, FaDu, SCC25, UMSCC2, and UMSCC74B) were authenticated by STR and all cell lines tested for mycoplasma contamination in the Translational Core Facility of the University of Maryland Marlene and Stewart Greenebaum Cancer Center. All cells were maintained in Dulbecco’s modified Eagle’s medium (DMEM) supplemented with 10% fetal bovine serum (FBS), 2 mM glutamine, and 100 U/mL penicillin and streptomycin (Gibco).

### Tumor lysates of patient-derived xenografts

Tumor lysates from two Patient-Derived Xenografts (PDX) were provided by Dr. Rena Lapidus, in the Translational Core Facility of the University of Maryland Marlene and Stewart Greenebaum Cancer Center. These PDX originated from NCI and were amplified in mice at the University of Maryland. The patient ID for the cisplatin-sensitive PDX is 784,116 and the cisplatin-resistant PDX is 871,537.

### Antibodies and reagents

The following antibodies were purchased from Cell Signaling Technology: ETS-1 (CST-14069), phospho-ERK (CST-4370), ERK (CST-4348), phospho-SRC-Y416 (CST-2101), SRC (CST-2123), cleaved caspase-3 (CST-9664), and β-actin (CST-4967). Phospho-ETS-1-T38 was from Abcam (ab59179) and Thermo Scientific (44-1104G). Cisplatin was purchased from Sigma (P4394). MEK inhibitor, PD0325901, and SRC inhibitors, Dasatinib and Saracatinib, were from Selleck Chemicals. Protease and phosphatase inhibitors were from Roche.

### Cell lysis and Western blot analysis

Cells were lysed and immunobotted as described previously [16, 17]. As needed, experiments were repeated for three times and densitometric analyses of Western blots were performed using ImageJ software.

### siRNA knockdown

Nonspecific control siRNAs and an siRNA SMARTpool ETS-1 were purchased from Dharmacon. An additional siRNA ETS-1 was purchased from Sigma using previously published sequences [18]. Cells were transfected with siRNA ETS-1 or nonspecific control siRNAs using DharmaFECT 1 reagent (Dharmacon) or Lipofectamine Rnaimax Transfection Reagent (Thermo Scientific) according to the manufacturer’s instructions.

### In vitro migration and invasion

For xCELLigence real-time migration and invasion experiments, cells were grown in 10 cm dishes using DMEM/10% FBS until we achieved approximately 60–80% confluence. xCELLigence CIM plates were pre-coated with matrigel diluted 1:10 with serum-free media, according to the manufacturer’s instructions, for invasion studies and incubated at 37 °C for 4 h. For both migration and invasion experiments, CIM plates were assembled with either serum-free or complete media in bottom chambers, and serum-free media in top chambers, followed by an additional 1 h incubation for membrane equilibration. Cells were then washed, trypsinized, counted, and centrifuged at 300 x g for 10 min. Cell pellets were resuspended in serum-free media up to a concentration of 1 × 10^6^ cells/ml, and 50 μl of suspension was added to each upper chamber well. Plates were incubated at room temperature for 30 min before being placed into the RTCA system and incubated at standard conditions for 72 h.

### Cell growth assays

Cell growth was assessed by MTS assay using the CellTiter 96 Aqueous ONE Solution kit (Promega) as described previously [17, 19]. Briefly, cells (5 × 10^4^ cells/mL) were seeded into 96-well plates for 24 h. The next day, the media were replaced with fresh media that contained the indicated concentrations of cisplatin, PD0325901, a combination of cisplatin and PD0325901, or vehicle control (DMSO). After an additional 72 h of incubation, MTS reagent (20 μL) was added to each well and cells with the reagent were incubated at 37 °C for 2 h. Absorbance at 490 nm was measured using a microplate reader (Bio-Rad). Each experiment was performed in triplicate. The combination index values were determined according to the Chou–Talalay method [20] using CalcuSyn software.

### Colony formation assay

Cells (800–1000) transfected with siRNA control or ETS-1 for 24 h were seeded in 12-well plates and grown in normal media for 10 to 14 days, washed once with 1x PBS, fixed with methanol, and stained with crystal violet.

### Measuring apoptosis by Annexin V/propidium iodide staining

Cells were trypsinized, washed once with PBS and Annexin V binding buffer, and re-suspended in 1 mL Annexin V binding buffer. 2 × 10^5^ cells were then stained with 0.5 μL of Annexin V and 0.7 μL of propidium iodide (PI) for 15 min at room temperature. Staining was then analyzed by flow cytometry on the BD FACSCanto II™ Cell Analyzer (BD Biosciences). We used FCS Express 6 to analyze data. Experiments were performed in triplicate and statistical analysis was performed.

### Statistical analysis

Data are presented as mean ± SD. Statistical analysis was performed using GraphPad Prism version 7.04 (GraphPad Software, Inc.). *P* values < 0.05 were considered to be statistically significant (*P < 0.05; **P < 0.01; ***P < 0.005).

## Results

### ETS-1 is up-regulated in a majority of cisplatin-resistant HNSCC

**Fig. 1:**
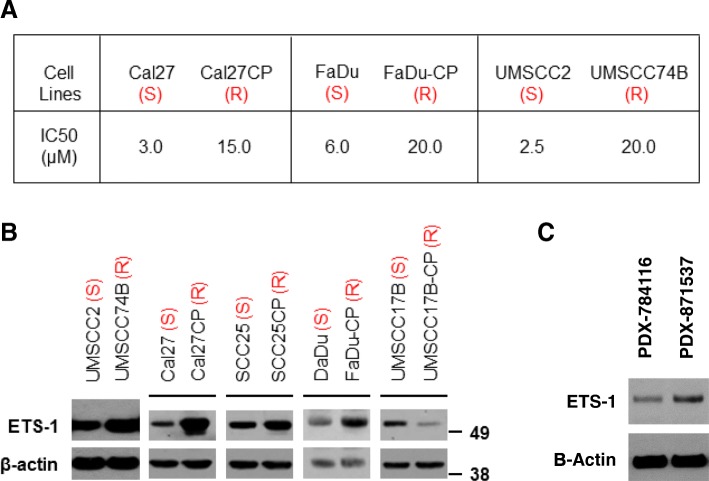
ETS-1 increases in cisplatin resistant HNSCC cells. **a**. IC_50_ values to cisplatin in the cisplatin-sensitive and resistant HNSCC cells are listed. Cells were treated with increasing doses of cisplatin for 72 h and IC_50_ values were determined. **b***.* ETS-1 protein levels were examined in the indicated cells by Western blot analysis. The experiments were repeated for three times. Note: (S) indicates sensitivity to cisplatin and (R) indicates resistance to cisplatin. *C.* ETS-1 expression levels were examined in cisplatin-sensitive (Patient ID: 784116) and resistant (Patient ID: 871537) PDX by Western blot

We previously reported two pairs of cisplatin-sensitive/resistant HNSCC cells: SCC25/ SCC25CP cells and UMSCC17B/UMSCC17B-CP cells [16]. In order to establish additional cisplatin-resistant HNSCC cell lines for our studies, we treated Cal27 and FaDu cells with 0.5 μΜ to 5 μΜ of cisplatin for 6 months before these cells were stably grown in media with 5 μΜ cisplatin. We then determined the IC_50_ of the parent cells (Cal27 and FaDu cells) and the cisplatin-treated cells (Cal27CP and FaDu-CP) by MTS assay. The results showed that the IC_50_ values of Cal27 and FaDu cells was 3 μΜ and 6 μΜ respectively, while those in Cal27CP and FaDu-CP were 15 μΜ and 20 μΜ respectively. These data suggested that the Cal27CP and FaDu-CP cells were cisplatin-resistant (Fig. 1a). We recently received UMSCC2 and UMSCC74B cells from Dr. Thomas E. Carey (University of Michigan, Ann Arbor, MI, USA). The UMSCC2 cells were from a patient untreated with cisplatin, whereas the UMSCC74B cells were from a patient treated with chemotherapy [21]. MTS assay showed that the IC_50_ of UMSCC2 cells was 2.5 μΜ while that of UMSCC74B cells was 20 μΜ, which indicated that the UMSCC2 cells were sensitive to cisplatin, whereas the UMSCC74B cells were resistant (Fig. 1a).

Next, we determined ETS-1 protein levels in the 5 cell pairs. ETS-1 protein level in patient derived UMSXCC74B cells was much higher than that of UMSCC2 cells (Fig. 1b). The three cisplatin-resistant HNSCC cells, including Cal27CP, SCC25CP, and FaDu-CP, also showed much higher expression of ETS-1 compared to their parental partner cells, whereas UMSCC17B-CP showed lower ETS-1 expression in comparison to UMSCC17B cells (Fig. 1b). To confirm the results from culture cells, we wanted to examine if ETS-1 expression in cisplatin-resistant head and neck cancer tissue is higher than that in cisplatin-sensitive tissues. Tumor lysates from two patient-derived xenografts (PDX) were acquired from a patient who was not treated with cisplatin prior to surgery and a patient treated with cisplatin before surgery. The results showed that the ETS-1 expression in cisplatin-resistant HNSCC was much higher than that in cisplatin-sensitive tissue (Fig. 1c). Our results indicated that ETS-1 protein levels were up-regulated in a majority of cisplatin-resistant HNSCC.

### ETS-1 regulates cell growth of cisplatin-resistant HNSCC

**Fig. 2:**
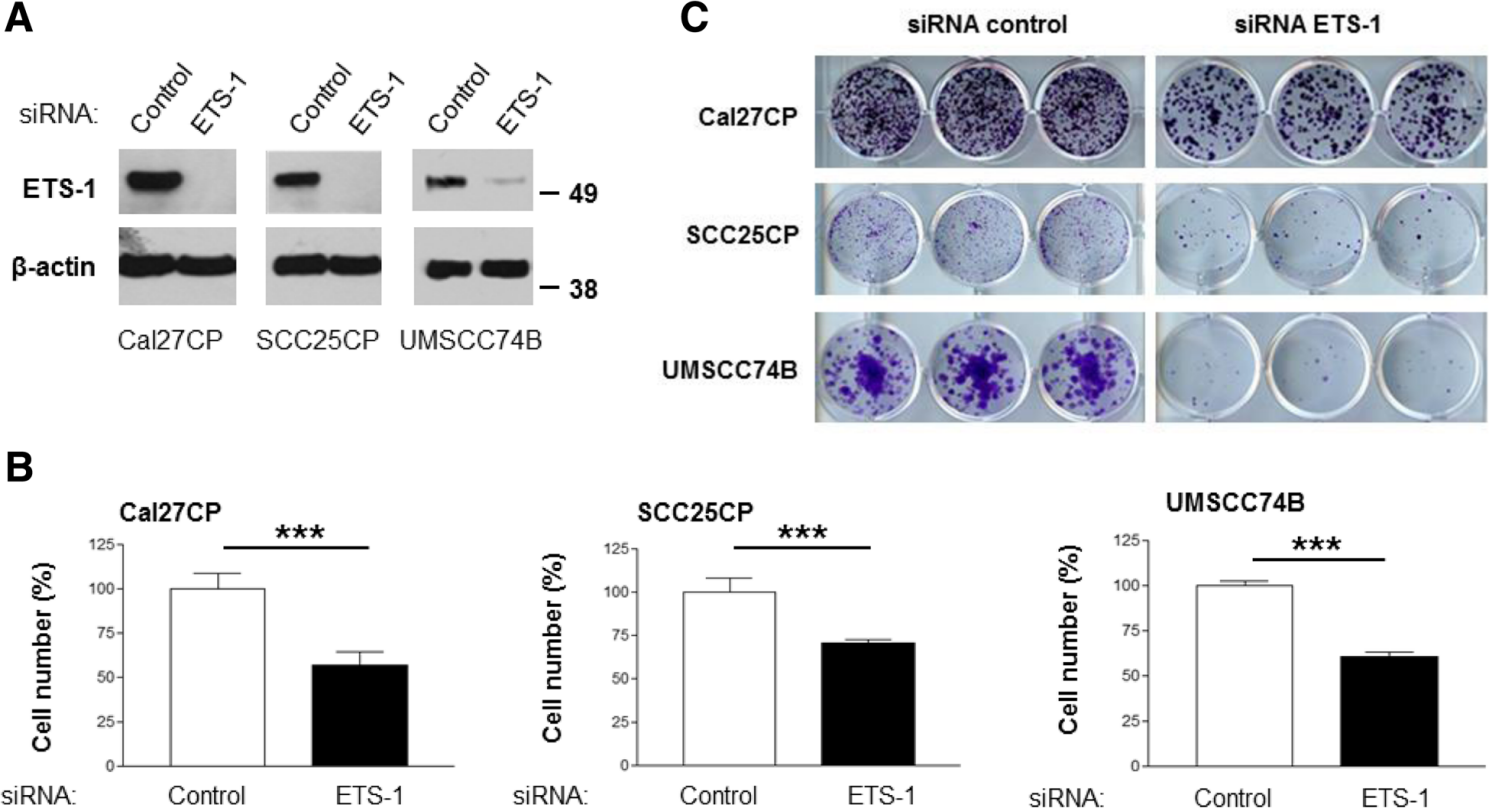
ETS-1 is crucial for cisplatin-resistant HNSCC cell proliferation. **a**. Cal27CP, SCC25CP, and UMSCC74B cells were transfected with non-target siRNA or siRNA against ETS-1 for 48 h and ETS-1 protein levels were detected by Western blot. **b**. Cells transfected with non-target siRNA or siRNA against ETS-1 for 24 h were split into 6-well plates, followed by growth for an additional 72 h, at which point cell numbers were counted. **c***.* Cells transfected with non-target siRNA or siRNA against ETS-1 for 24 h were split into 12-well plates (100 cells/well) and colony formation was observed after 7 days

A previous study by Liu, et al.*,* [18] showed that knockdown of ETS-1 by a siRNA against ETS-1 blocked the signaling and function of platelet-derived growth factor D-chain (PDGF-D). Therefore, we wanted to determine if ETS-1 also played a role in cisplatin-resistant HNSCC growth by using the same ETS-1 siRNA. ETS-1 expression was effectively knocked down in Cal27CP, SCC25CP, and UMSCC74B cells by ETS-1 siRNA (Fig. 2a). The number of cells in ETS-1 knockdown samples was less than control samples three days after siRNA transfection (Fig. 2b). Next, the same number of cells transfected with non-target siRNA or siRNA against ETS-1 was seeded in 12-well plates for the colony formation assay. We found that ETS-1 knockdown completely blocked colony formation of UMSCC74B cells and significantly decreased colony formation of Cal27CP SCC25CP cells (Fig. 2c). In order to confirm the above results, we used another siRNA against ETS-1 (siRNA SMARTpool human ETS-1, L-003887, Dharmacon) to knock down ETS-1 in Cal27CP cells. Consistent with previous data, this siRNA also successfully knocked down ETS-1 expression and decreased cell proliferation (data not shown). Taken together, our data demonstrated that ETS-1 was crucial for cisplatin-resistant HNSCC proliferation.

The significant basal levels of ETS-1 in cisplatin-sensitive HNSCC lines such as SCC25 and Cal27 cell suggested that ETS-1 may also play a role in the regulation of cisplatin-sensitive HNSCC cell growth. Indeed, we found that knockdown of ETS-1 in SCC25 Cal27 cells also led to inhibition of cell growth in these cells (Additional file 1: Figure S1A and Figure S1B). These data indicate that ETS-1 played an important role in both cisplatin-sensitive and resistant HNSCC cells, although the current study mainly focused on cisplatin-resistant HNSCC cells.

### ETS-1 regulates cell migration and invasion of cisplatin-resistant HNSCC

**Fig. 3:**
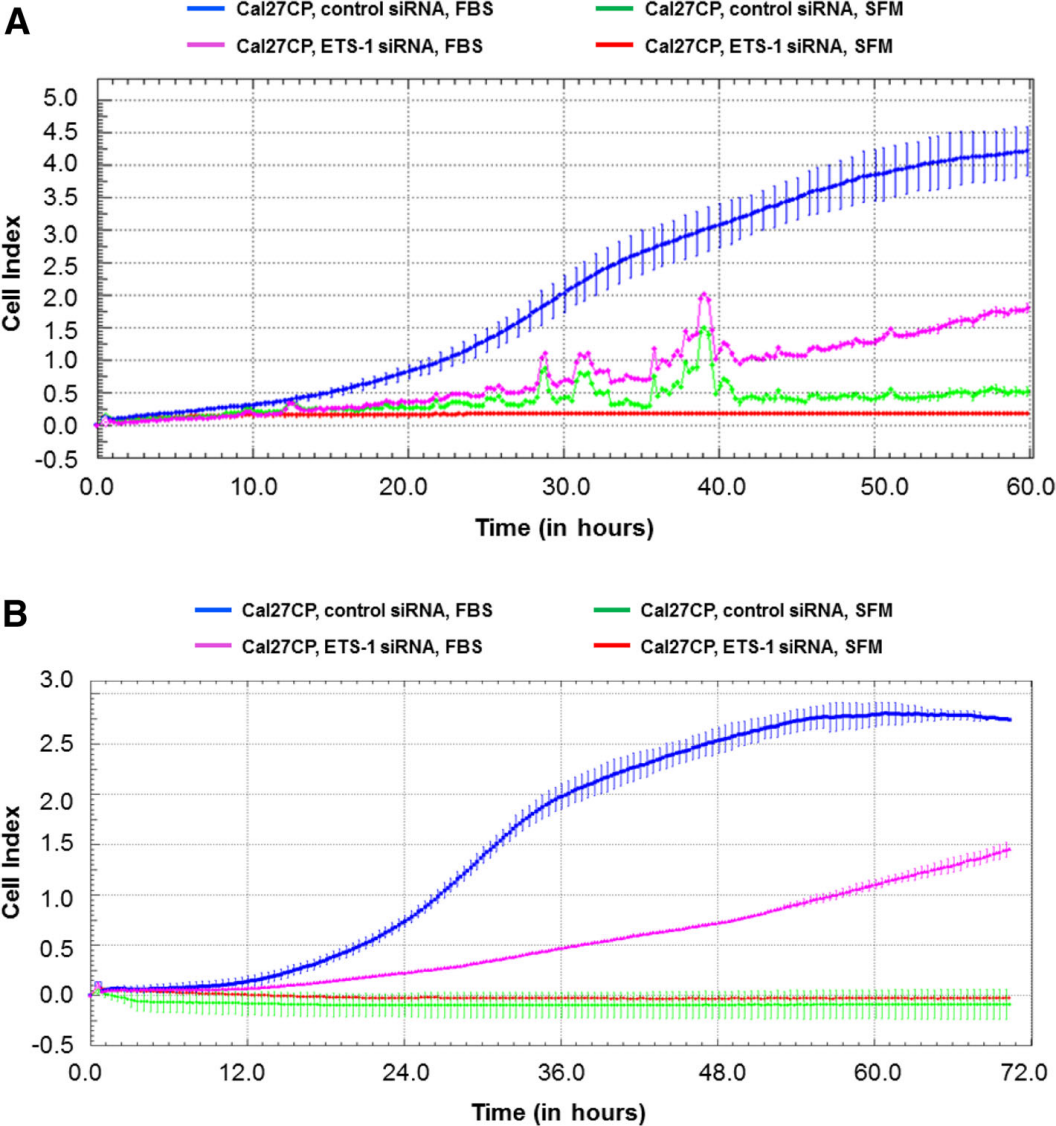
ETS-1 is important in regulation of cisplatin-resistant HNSCC cell migration and invasion. Cal27CP cells were transfected with non-target siRNA or siRNA against ETS-1 for 48 h and cell migration (**a**) and invasion (**b**) were monitored with the xCELLigence real-time cell system. SFM: serum-free media

We next examined whether migration and invasion were regulated by ETS-1. Cal27CP were transfected with ETS-1 siRNA and cell migration was monitored by the xCELLigence real-time cell system for 48 h post-transfection. Cell migration was significantly impaired upon ETS-1 knockdown (Fig. 3a). In addition, knockdown of ETS-1 also significantly impaired Cal27CP invasion (Fig. 3b). In line with these results, ETS-1 knockdown slowed cell migration and invasion in UMSCC74B cells (Additional file 1: Figure S2A and Figure S2B). Knockdown of ETS-1 also significantly impaired cisplatin sensitive Cal27 migration and invasion (Additional file 1: Figure S3A and Figure S3B). Taken together, our data indicated that ETS-1 played an important role in the regulation of cell migration and invasion in both cisplatin-sensitive and resistant HNSCC.

### ETS-1 is associated with cisplatin resistance

**Fig. 4:**
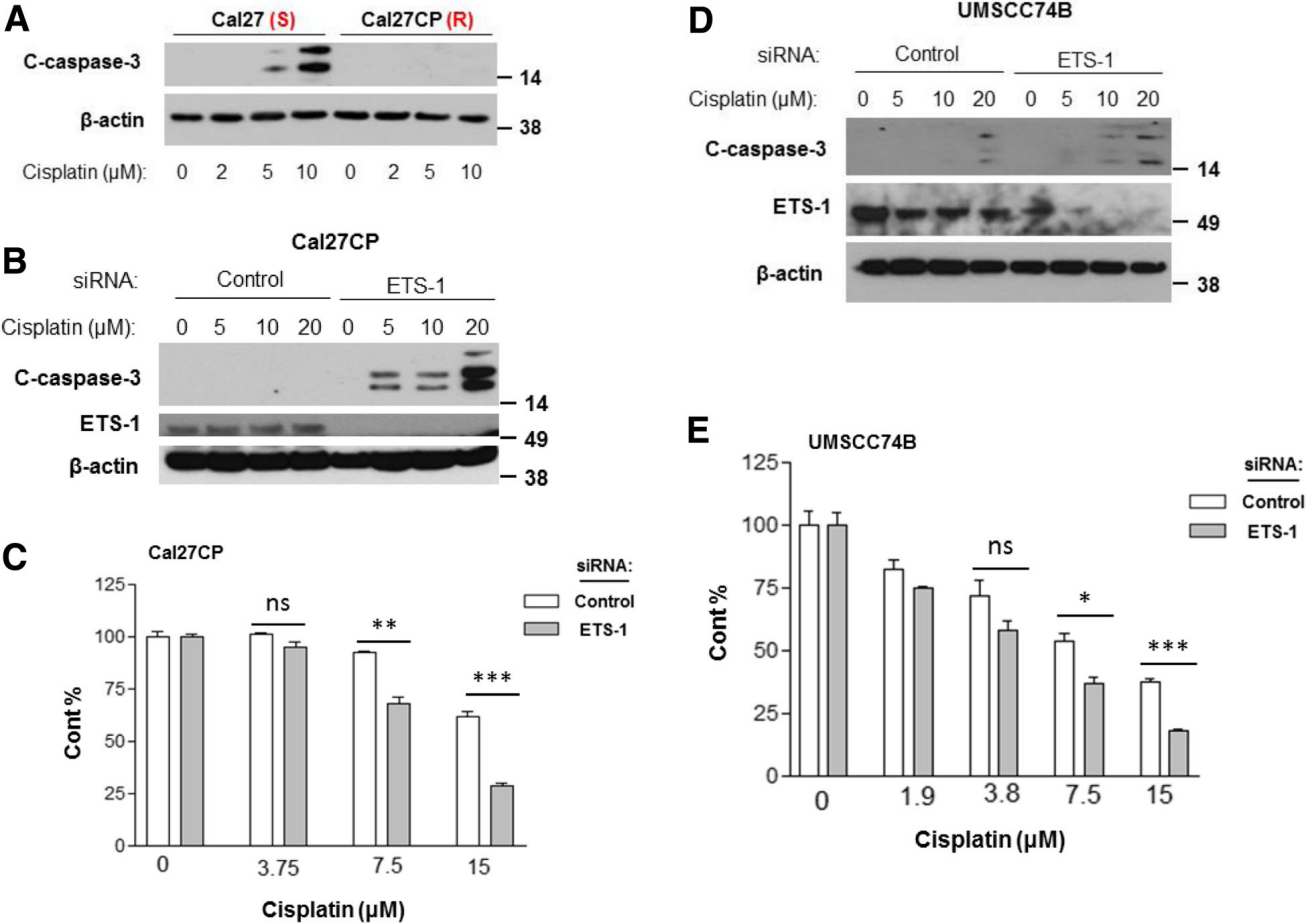
ETS-1 is involved in cisplatin resistance in HNSCC. **a**. Cal27 and Cal27CP cells were treated with increasing doses of cisplatin for 24 h, lysed, and analyzed for caspase-3 cleavage by Western blot with β-Actin as loading control. **b** and **d**. Cal27CP (*B*) and UMSCC74B (**d**) cells were transfected with non-target siRNA or siRNA against ETS-1 for 24 h, treated with increasing doses of cisplatin for 24 h, and the expression of the indicated proteins was detected by Western blot. *C* and *E*. Cal27CP (**c**) and UMSCC74B (**e**) cells were transfected with non-target siRNA or siRNA against ETS-1 for 24 h, seeded into 6-well plates for 24 h, then treated with increasing doses of cisplatin for 72 h. Cell proliferation was measured by MTS assay

We next determined whether ETS-1 knockdown could re-sensitize cisplatin-resistant HNSCC to cisplatin treatment. We first treated Cal27 and Cal27CP cells with different doses of cisplatin (2, 5, or 10 μΜ) for 24 h and checked caspase-3 levels in these cells by Western blot analysis. Dose-dependent caspase-3 cleavage was detected in Cal27 cells, but not in Cal27CP cells after cisplatin treatment (Fig. 4a). These results suggested that Cal27CP cells are cisplatin-resistant. Next, Cal27CP cells transfected with either siRNA control or siRNA ETS-1 were treated with different doses of cisplatin for 24 h before caspase-3 cleavage was detected by Western blot analysis. In the control cells treated, induction of caspase-3 cleavage did not occur, whereas in siRNA ETS-1 transfected cells, cisplatin induced caspase-3 cleavage in a dose-dependent manner (Fig. 4b). MTS assay results showed that ETS-1 knockdown synergized with cisplatin to inhibit Cal27CP cell growth (Fig. 4c). Only a high dose (20 μΜ) of cisplatin induced caspase-3 cleavage in UMSCC74B cells treated with siRNA control, whereas 10μΜ of cisplatin was able to induce caspase-3 cleavage in UMSCC74B transfected with siRNA ETS-1 (Fig. 4d). Moreover, XTT assay data also showed that ETS-1 knockdown re-sensitized UMSCC74B cells to cisplatin treatment (Fig. 4e). These data suggested that ETS-1 is involved in cisplatin resistance in HNSCC.

### MEK/ERK pathway does not regulate ETS-1 in cisplatin-resistant HNSCC

**Fig. 5:**
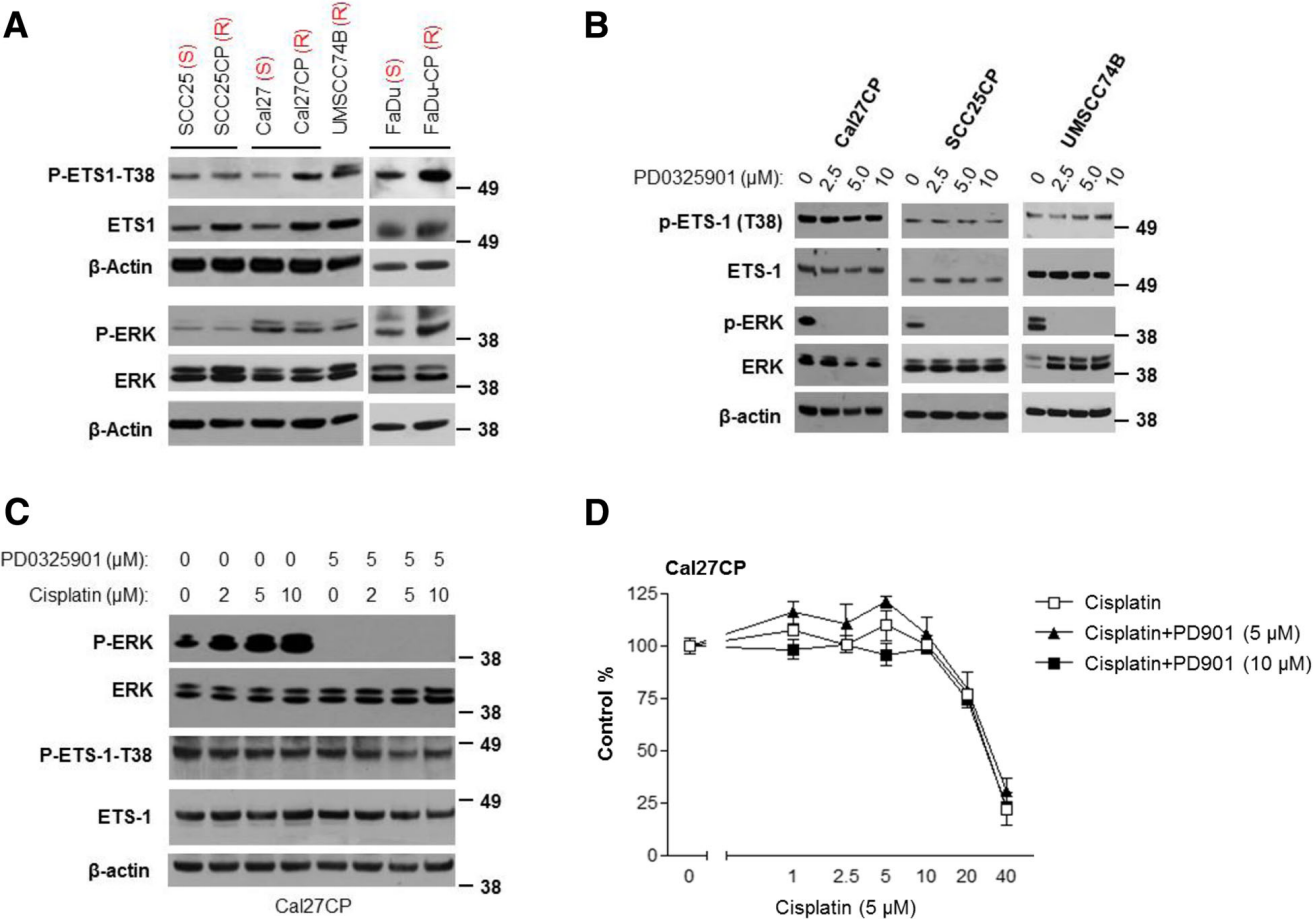
ETS-1 is not controlled by the MEK/ERK pathway and inhibition of the MEK/ERK pathway does not re-sensitize cisplatin-resistant HNSCC cells to cisplatin treatment. **a**. Cell lysates were prepared from SCC25/SCC25CP, Cal27/Cal27CP, and FaDu/FaDu-CP cell pairs, as well as UMSCC74B cells. Phosphorylation and total levels of ERK and ETS-1, as well as β-actin, were detected by Western blot. **b**. Cells were treated with different doses of PD0325901 for 24 h, lysed, and phosphorylation of ERK and ETS-1-T38 and total levels of ERK, ETS-1, and β-actin were detected by Western blot. **c**. Cal27CP cells were treated with different doses of cisplatin alone or in combination with PD0321901 (5 μM) for 24 h, and phosphorylation of ERK and ETS-1-T38 and total levels of ERK, ETS-1 and β-actin were detected by Western blot. **d***.* Cal27CP cells were treated with DMSO control, cisplatin, PD0325901, or a combination for 72 h. Cell proliferation was measured by MTS assay

Previous studies reported that the MEK/ERK pathway regulated ETS-1 through ERK phosphorylation of ETS-1 threonine 38 (T38) in many types of cancers [5, 10–14]. Our data demonstrated that, consistent with ETS-1 expression, cisplatin-resistant HNSCC cells (SCC25CP, Cal27CP and FaDu-CP cells) had higher levels of ETS-1 phosphorylation at T-38 compared to their parental cells (Fig. 5a). However, although ERK phosphorylation in FaDu-CP was higher than that in their parental partner FaDu cells, phosphorylation of ERK in SCC25CP and Cal27CP cells was similar to those in their parental partners (Fig. 5a). These results prompted us to further examine whether or not MEK/ERK pathways could control ETS-1-T38 phosphorylation in cisplatin-resistant HNSCC. Cal27CP cells were treated with PD0325901, a MEK inhibitor [22–25], for 24 h, followed by detection of ERK and ETS-1-T38 phosphorylation. The results showed that treatment with a MEK inhibitor could completely block phosphorylation of ERK but did not decrease ETS-1-T38 phosphorylation (Fig. 5b, left panel). In addition, the MEK inhibitor had no effect on ETS-1 expression (Fig. 5b, left panel). Similar results were found in SCC25CP (Fig. 5b, middle panel) and UMSCC74B (Fig. 5B, right panel) cells. Our data indicate that (i), ETS-1 is not a downstream target of MEK/ERK pathways in cisplatin-resistant HNSCC, and (ii), other potential kinases can phosphorylate ETS-1-T38 in these cells.

We further investigated whether inhibition of MEK/ERK pathways could re-sensitize cisplatin-resistant HNSCC to cisplatin treatment. Cal27CP cells were treated with different doses of cisplatin alone or in combination with PD0325901 for 24 h. Cisplatin induced dose-dependent ERK phosphorylation, which PD0325901 treatment completely blocked, but ETS-1 phosphorylation was neither elevated by cisplatin nor inhibited by PD0325901 (Fig. 5c). In addition, neither treatment, alone or in combination, could induce caspase-3 cleavage (data not shown). Similar results were found in UMSCC74B cells (Additional file 1: Figure S4A). Furthermore, we demonstrated that a combination with PD0325901 did not improve the efficacy of cisplatin on the inhibition of cell proliferation by MTS assay in Cal27CP (Fig. 5d) and UMSCC74B cells (Additional file 1: Figure S4B). Our data indicated that inhibition of MEK/ERK pathway did not re-sensitize cisplatin-resistant HNSCC to cisplatin treatment.

### SRC kinase controls ETS-1 in cisplatin-resistant HNSCC

**Fig. 6:**
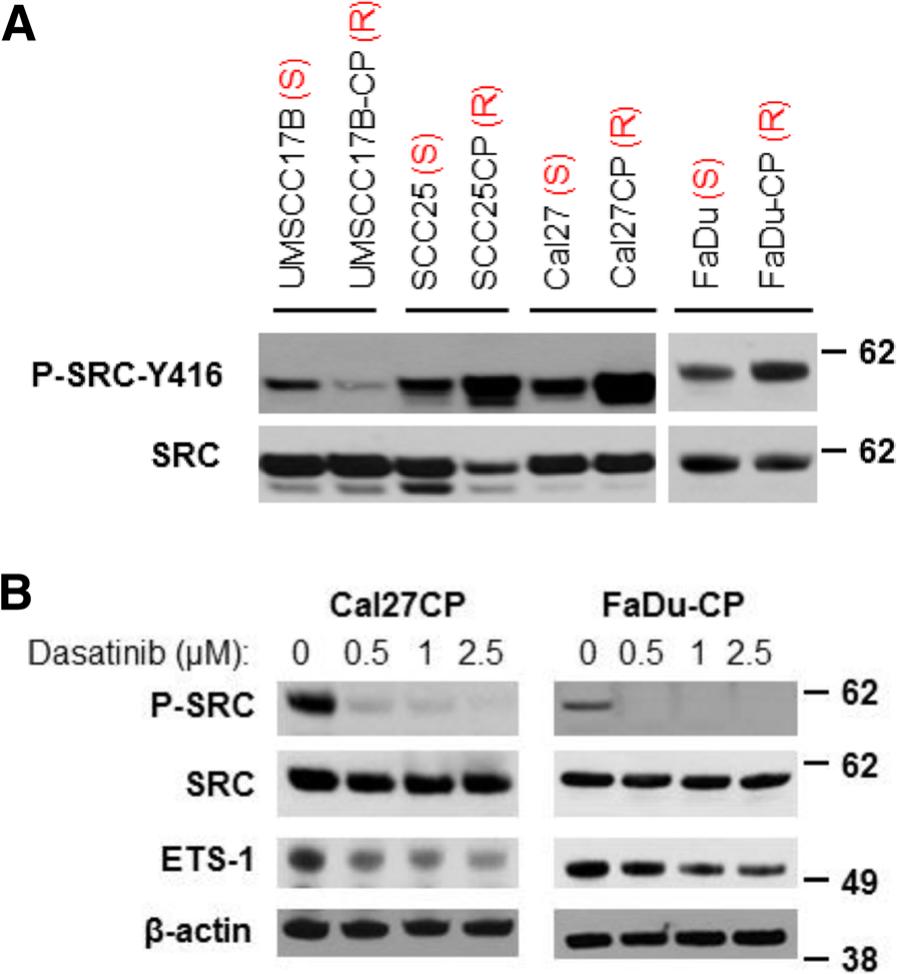
ETS-1 is regulated by SRC. **a**. Cell lysates prepared from Fig. 1b were re-used to test phosphorylation and total SRC by Western blot analysis. **b**. Cells were treated with different doses of Dasatinib for 8 h, lysed, and phosphorylation and total SRC and ETS-1 levels were detected by Western blot

It has been reported that SRC is activated and regulates tumorigenesis through phosphorylation of ETS-1 in breast cancer [5, 15]. Therefore, we examined if the SRC/ETS-1 signaling pathway was upregulated in cisplatin-resistant HNSCC. As shown in Fig. 6a, phosphorylation of SRC at tyrosine 417 (SRC-Y-417) in SCC25CP, Cal27CP and FaDu-CP cells was dramatically higher than that in their parental cells (Fig. 6a), while phosphorylation of SRC in UMSCC17B-CP cells was lower than that in its parental cell, UMSCC17B. These data indicate that SRC activity in the majority of cisplatin-resistant HNSCC was elevated (Fig. 6a). Combined with the results in Fig. 1 that showed up-regulation of ETS-1 protein levels in a majority of cisplatin-resistant HNSCC cells, we concluded that the SRC/ETS-1 pathway was also enhanced in a majority of cisplatin-resistant HNSCC (Fig. 6a). Next, we treated Cal27CP and FaDu-CP cells with increasing doses of the SRC inhibitor, Dasatinib, and examined SRC phosphorylation and ETS-1 expression. Dasatinib significantly blocked phosphorylation of SRC and led to dose-dependent decreases of ETS-1 expression in these cells (Fig. 6b). These results indicate that SRC regulates ETS-1, and SRC/ETS-1 signaling is up-regulated in cisplatin-resistant HNSCC.

### Dasatinib suppresses proliferation, migration, and invasion in cisplatin-resistant HNSCC

**Fig. 7:**
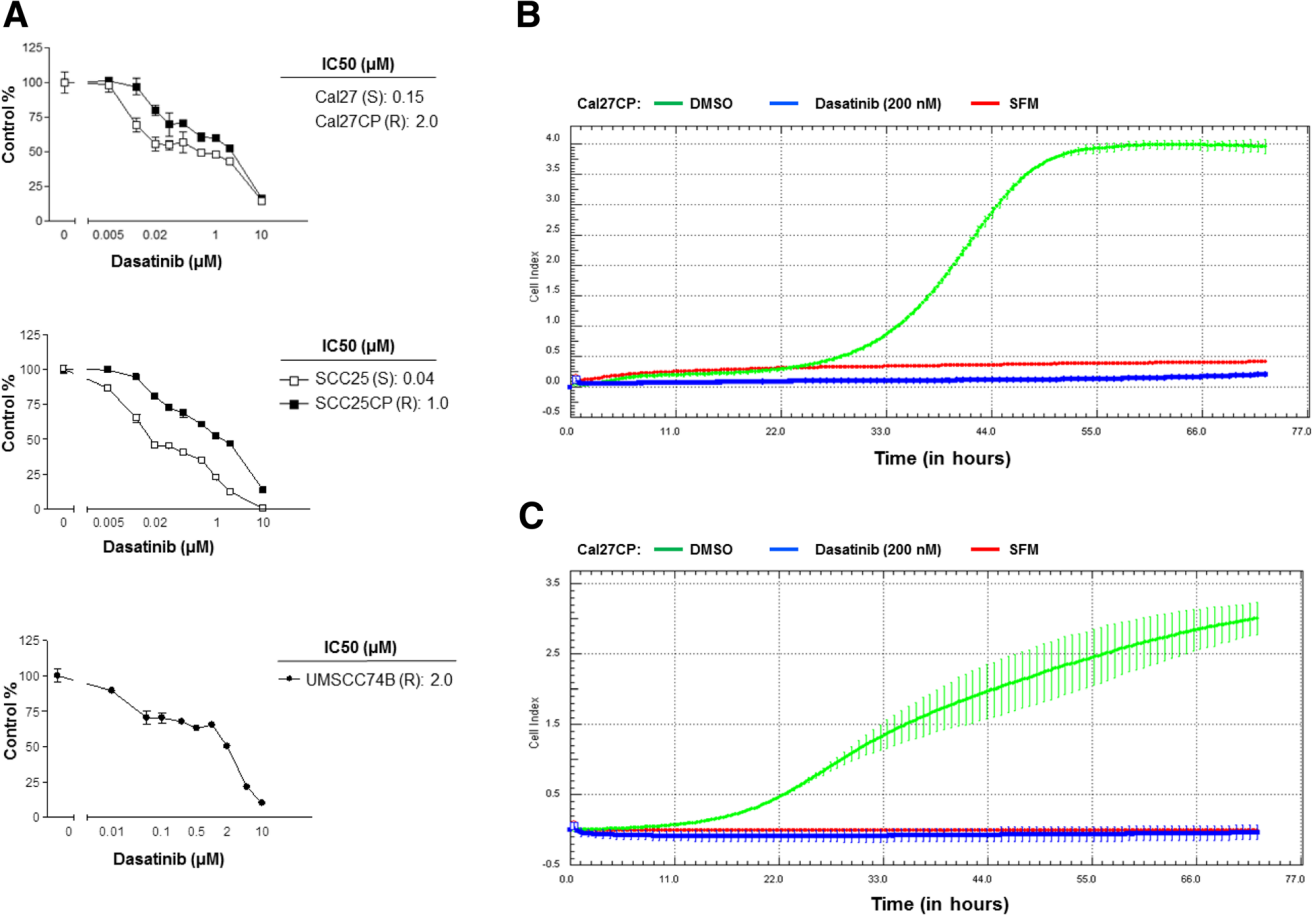
Inhibition of cisplatin-resistant HNSCC by SRC inhibitor, Dasatinib. **a***.* Cells were treated with increasing doses of Dasatinib for 72 h and IC_50_ values were determined. **b** and **c**. Cal27CP cells were treated with DMSO control or 200 nM Dasatinib and cell migration (**b**) and invasion (**c**) were monitored by the xCELLigence real-time cell system. Note: SFM: serum-free media

Previous studies demonstrated that Dasatinib significantly inhibited HNSCC cell proliferation and survival [26–28]. We next determined whether Dasatinib could inhibit cell proliferation in both cisplatin-sensitive and resistant HNSCC. Dasatinib inhibited SCC25CP, Cal27CP, and UMSCC74B proliferation in a dose-dependent manner with an IC_50_ of 1.0 μΜ for SCC25CP cells and 2.0 μΜ for both Cal27CP and UMSCC74B cells. Meanwhile, it inhibited SCC25 and Cal27 proliferation in a dose-dependent manner with an IC_50_ of 0.04 μΜ for SCC25 cells and 0.15 μΜ for Cal27 cells, which indicated that cisplatin-resistant HNSCC were relatively resistant to Dasatinib (Fig. 7a). Moreover, treatment with 200 nM of Dasatinib led to complete inhibition of cell migration (Fig. 7b) and invasion in Cal27CP cells (Fig. 7c). In addition, we also found that treatment with 1–2 μΜ Dasatinib completely inhibited cell migration and invasion in parental Cal27 cells (Additional file 1: Figure S5A and Figure S5B). Our data indicated that the SRC inhibitor, Dasatinib could suppress cell proliferation, migration, and invasion in both cisplatin-sensitive and resistant HNSCC cells, although cisplatin-resistant HNSCC cells remained relatively resistant to Dasatinib.

### Dasatinib cooperates with cisplatin to induce apoptosis in cisplatin-resistant HNSCC

**Fig. 8:**
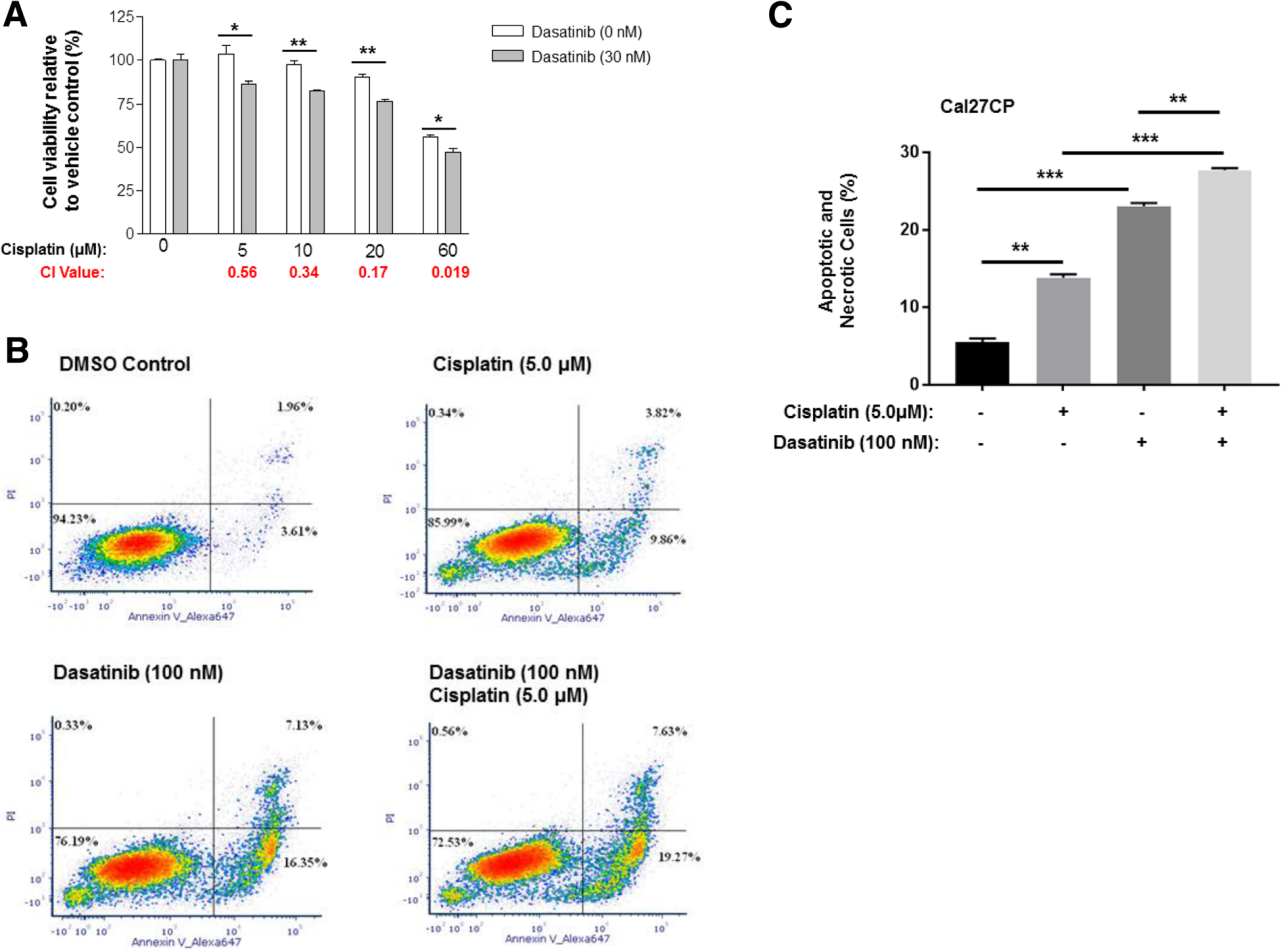
Dasatinib cooperates with cisplatin to inhibit cell proliferation and induce apoptosis in cisplatin-resistant HNSCC. **a***.* Cells were treated with DMSO control, cisplatin, Dasatinib, or a combination for 72 h. Cell proliferation was measured by MTS assay. Each experiment was performed in triplicate. The combination index values were determined according to the Chou–Talalay method. **b***.* Cells were treated with DMSO control, cisplatin, Dasatinib, or a combination for 48 h. Cell apoptosis was measured by Annexin V staining and flow cytometry analysis. Experiments were performed in triplicate and representative flow cytometry results from each treatment were shown and the percentages of early apoptotic cells (lower right), late apoptotic cells (upper right), cells of necrosis (upper left), and live cells (lower left) were indicated, respectively. **c**. Statistical analysis of results in **b** was performed. *P* values < 0.05 were considered to be statistically significant. Note: *P < 0.05, **P < 0.01, ***P < 0.005

Next, Cal27CP cells were treated with different doses of cisplatin alone or in combination with Dasatinib (30 nM). Cisplatin modestly inhibited cell proliferation, but when combined with Dasatinib, displayed increased inhibition compared to treatment with cisplatin alone (Fig. 8a). In order to determine whether cisplatin and Dasatinib synergistically suppress cell proliferation, we used CalcuSyn software to analyze the combination index values (CI) according to the Chou–Talalay method [20]. CI values from all combined inhibitor doses were less than 1 (Fig. 8a). These results demonstrated a strong synergism between Dasatinib and cisplatin in Cal27CP cells. Similar results were found in SCC25CP cells (Additional file 1: Figure S6A). Furthermore, Dasatinib induced dramatic apoptosis while cisplatin induced apoptosis to a lesser degree, but the combination of Dasatinib and cisplatin led to significantly greater apoptosis compared to either treatment alone in Cal27CP (Fig. 8b and c) and SCC25CP cells (Additional file 1: Figure S6B and Figure S6C). These data suggest that Dasatinib and cisplatin cooperate to induce apoptosis.

## Discussion

In this study, we examined the expression of transcription factor ETS-1 and determined its functions and regulation in cisplatin-resistant HNSCC cells. We found that ETS-1 increased in multiple cisplatin-resistant HNSCC cell lines, including one derived from patients (UMSCC74B). Moreover, we showed that ETS-1 knockdown led to inhibition of cell proliferation, migration, and invasion. In addition, ETS-1 knockdown re-sensitized cells to cisplatin treatment. Furthermore, we showed that ETS-1 promoted tumorigenesis downstream of the SRC pathway independent of the MEK/ERK pathway. Our data indicated that SRC/ETS-1 signaling could be a potential therapeutic target for cisplatin-resistant HNSCC.

Previous studies showed that silencing ETS-1 increased cell proliferation in vitro and reduced tumor growth in vivo in some breast cancer cell lines [5, 28, 29]. Conversely, silencing ETS-1 suppressed cell growth of human renal carcinoma (786–0) and human glioma (U87MG) cell lines both in vitro and in vivo [30]*.* In addition, researchers described that ETS-1 promoted cell proliferation through induction of cyclin E, CDK2 (cyclin-dependent kinase 2), and c-Myc expression [31, 32]. Our data demonstrate that ETS-1 promotes cell proliferation in all cisplatin-resistant HNSCC cell lines tested. However, ETS-1 silencing was unable to decrease CDK2, Cyclin E, and c-Myc expression (data not shown). It remains of utmost importance to further define the downstream targets of ETS-1 that play a role(s) in regulation of cell proliferation in cisplatin-resistant HNSCC cells.

Our data indicate that ETS-1 is important to modulate cell migration and invasion. Many studies have shown that epithelial-to-mesenchymal transition (EMT) involves cancer migration, invasion, and metastasis. Two crucial EMT markers, which include up-regulated N-cadherin and down-regulated E-cadherin in cancer cells, play essential roles in EMT. Additionally, EMT is associated with other transcription factors, such as Snail, Slug, and Twist [33, 34]. We currently have no evidence for ETS-1 involvement in regulation of protein expression. Therefore, we did not discover what effect, if any, silencing of ETS-1 had on the expression of N-cadherin, E-cadherin, Snail, Slug, and Twist (data not shown). It would be interesting to identify the crucial proteins and signaling pathways that cooperate with ETS-1 to facilitate EMT in cisplatin-resistant HNSCC.

The roles of EST-1 in the regulation of cisplatin resistance have not been well-investigated. An earlier study by Wilson and colleagues showed that ETS-1 levels increased and conferred cisplatin resistance in ovarian cancer [35]. ETS-1 also regulated resistance to other therapies, including androgen receptor inhibitor and paclitaxel in prostate and breast cancers, respectively [36, 37]. Our data demonstrate that decreases in ETS-1 levels enhance the efficacy of cisplatin in inhibition of cell proliferation and survival. It should be noted that if ETS-1 can confer cisplatin resistance, it may be cancer- or cell-type specific.

Transcription and post-translation regulate ETS-1 expression and activity [5, 22]. Consistent with this, our data showed that a majority of cisplatin-resistant HNSCC cell lines expressed higher levels of ETS-1 protein when compared to their parental cells. These data imply that some crucial proteins and signaling pathways could regulate ETS-1 protein in all cisplatin-resistant HNSCC cells through post-translation mechanisms.

ETS-1 expression, stability, and activity are regulated by phosphorylation of multiple tyrosine kinases and serine/threonine kinases [5]. Notably, many studies have already demonstrated that MEK/ERK signaling pathways could control ETS-1 expression and activity through ERK phosphorylation of ETS-1 at Threonine 38 in many types of cancers [10–14]. Our data demonstrate that ETS-1 phosphorylation at T38 is unrelated to MEK/ERK pathways, which suggests that other potential kinases could phosphorylate ETS-1 at T38 in cisplatin resistant HNSCC. It is crucial to find the potential kinase that can phosphorylate ETS-1 at T38 and other sites in cisplatin-resistant HNSCC. This would, in turn, facilitate the search to find potential ETS-1 regulators and important therapeutic targets for cisplatin-resistant HNSCC treatment.

It has been recently reported that MEK/ERK pathways play crucial roles in the conferrance of drug resistance in multiple types of cancer [38–41]. Our data showed that MEK/ERK pathway inhibition did not re-sensitize cisplatin-resistant HNSCC to cisplatin treatment (Fig. 5). It is important to note that the role of MEK/ERK pathways in the regulation of cisplatin resistance could be also cell- or cancer-type specific. In addition, it is also possible that MEK/ERK signaling may regulate cisplatin resistance through ETS-1-independent mechanisms in a portion of cisplatin-resistant HNSCC. To this end, a study to determine whether MEK/ERK inhibitors synergistically enhance the efficacy of other anticancer drugs on inhibition of cell proliferation in cisplatin-resistant HNSCC is forthcoming.

Our results indicate that SRC kinase activity is elevated in cisplatin-resistant HNSCC, which is consistent with increased ETS-1 levels in multiple cisplatin-resistant HNSCC cell lines. Moreover, inhibition of SRC phosphorylation by Dasatinib caused dose-dependent decreases of ETS-1 levels. In addition, treatment of cisplatin-resistant HNSCC cells with Dasatinib alone dramatically suppressed cell proliferation, survival, migration, and invasion. Likewise, consistent with the knockdown of ETS-1 results, Dasatinib cooperated with cisplatin to inhibit cell proliferation. Our data suggest that SRC/ETS-1 is a crucial target in cisplatin-resistant HNSCC.

Previous studies have reported that Dasatinib shows significant anti-cancer activity in multiple cisplatin-sensitive HNSCC cell lines [26–28]. We noticed that Dasatinib displayed toxicity to both cisplatin-sensitive and resistant HNSCC cells, but the IC_50_ values for cisplatin-resistant HNSCC were relatively higher than that of cisplatin-sensitive HNSCC cells (Fig. 7a). It should be emphasized that the SRC/EST-1 signaling pathway could be a therapeutic target for both cisplatin-sensitive and resistant HNSCC, although our current study mainly focused on cisplatin-resistant HNSCC.

We also noticed that although combination of Dasatinib and cisplatin showed synergy, this combination could not completely suppress cisplatin resistant HNSCC cell proliferation (Fig. 7). In addition, given the fact that inhibition of a single pathway by an inhibitor alone is almost impossible to completely suppress tumor growth in vitro and in vivo, we are currently performing experiments to identify crucial survival pathways that are up-regulated under SRC/ETS-1 inhibition. This will ultimately facilitate subsequent studies to discover mechanism-based combination therapies, including Dasatinib, for cisplatin-resistant HNSCC.

## Conclusion

This study demonstrates that the up-regulation of the SRC-ETS1 survival pathway is involved in cell proliferation, survival, migration, invasion and resistance to cisplatin in head and neck squamous cell carcinoma. Inhibition of SRC by SRC inhibitor Dasatinib re-sensitizes cisplatin resistant head and neck squamous cell carcinoma to cisplatin treatment. Therefore, the SRC/ETS-1 pathway may be a key therapeutic target in cisplatin-resistant HNSCC treatment.

## Additional file

Additional file 1: **Figure S1.** ETS-1 is important for cisplatin sensitive HNSCC cell proliferation. **Figure S2.** ETS-1 is also important for UMSCC74B cell migration and invasion. **Figure S3.** ETS-1 is important for Cal27 cell migration and invasion. **Figure S4.** MEK/ERK inhibitor, PD0325901, does not re-sensitize cisplatin-resistant UMSCC74B cells to cisplatin treatment. **Figure S5.** Dasatinib inhibits cisplatin sensitive Cal27 cell migration and invasion. **Figure S6.** Dasatinib synergizes with cisplatin to induce apoptosis in SCC25CP cells. (DOCX 1215 kb)

## Abbreviations

CDK2: Cyclin-dependent kinase 2
CI: Combination index values
EMT: Epithelial-to-Mesenchymal Transition
ERK: Extracellular signal-regulated kinases
HNSCC: Head and Neck Squamous Cell Carcinoma
IC50: Half maximal inhibitory concentration
PDX: Patient-Derived Xenografts
