# Supplementary material for: Regulation of cisplatin-resistant head and neck squamous cell carcinoma by the SRC/ETS-1 signaling pathway
Source: BMC Cancer. 2019 May 22;19:485. doi: 10.1186/s12885-019-5664-7 (PMC6532223; doi:10.1186/s12885-019-5664-7)
Supplement: Supplementary file 1 — Figure S1. ETS-1 is important for cisplatin sensitive HNSCC cell proliferation. Figure S2. ETS-1 is also important for UMSCC74B cell migration and invasion. Figure S3. ETS-1 is important for Cal27 cell migration and invasion. Figure S4. MEK/ERK inhibitor, PD0325901, does not re-sensitize cisplatin-resistant UMSCC74B cells to cisplatin treatment. Figure S5. Dasatinib inhibits cisplatin sensitive Cal27 cell migration and invasion. Figure S6. Dasatinib synergizes with cisplatin to induce apoptosis in SCC25CP cells. (DOCX 1215 kb) [file 12885_2019_5664_MOESM1_ESM.docx]

**Supplementary Data:**

**Regulation of cisplatin-resistant head and neck squamous cell carcinoma by the SRC/ETS-1 signaling pathway**

**Supplementary Figure 1**

**
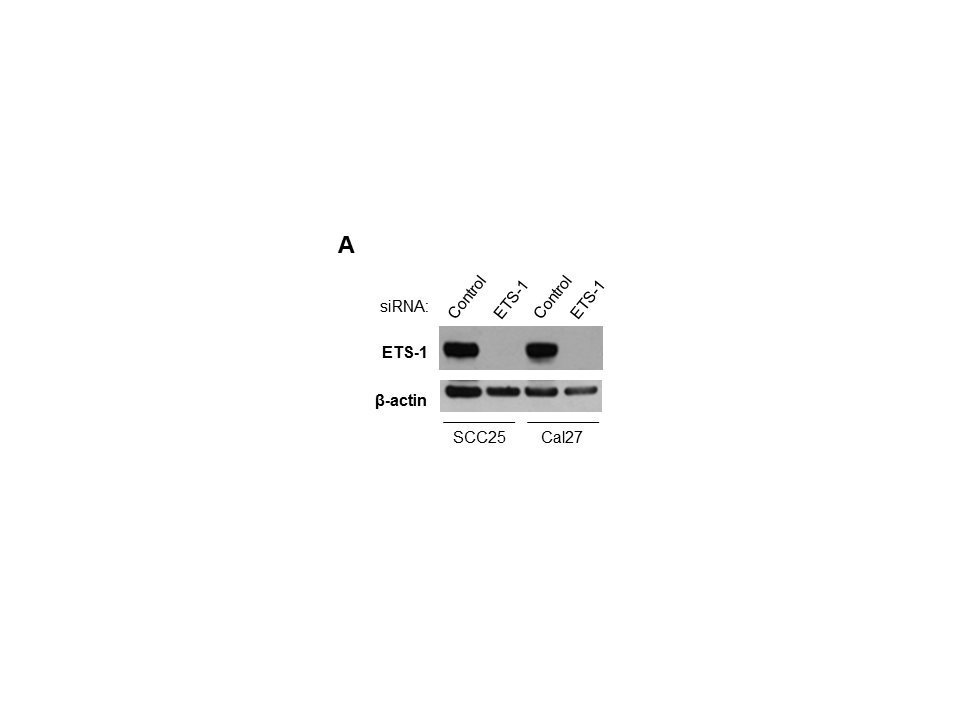
**

**
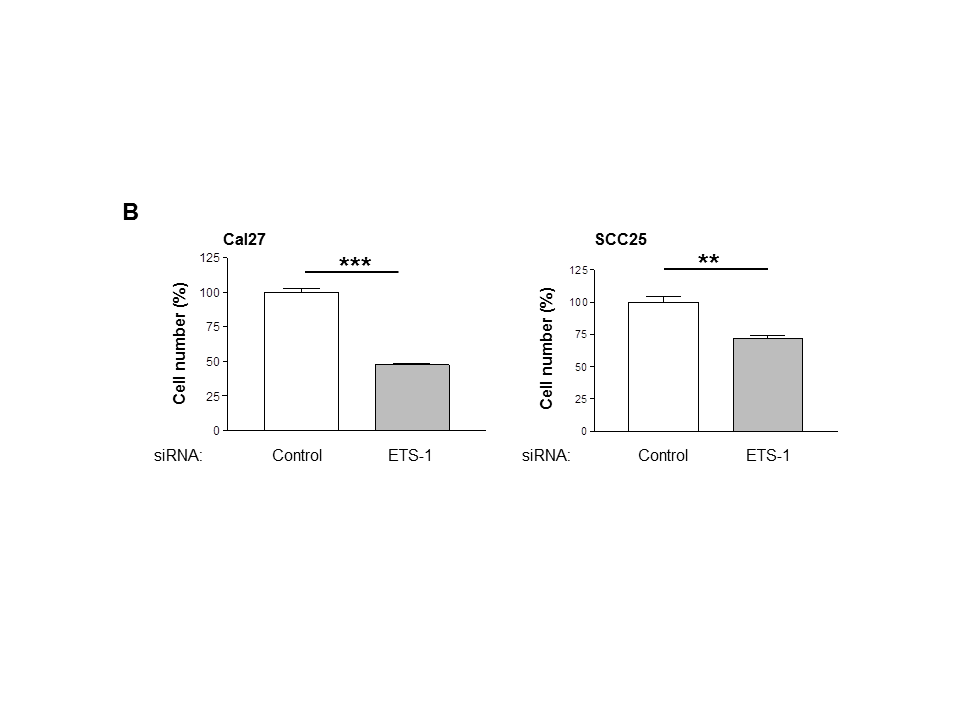
**

# Supplementary Figure 1 related to Figure 2.

# ETS-1 is important for cisplatin sensitive HNSCCC cell proliferation.

# *A*. Cells were transfected with non-target siRNA or siRNA against ETS-1 for 48 hours and the expression of ETS1 protein was detected by Western blot. *B*. Cells transfected with non-target siRNA or siRNA against ETS-1 for 24 hours were split into 6-well plates, followed by growth for an additional 72 hours when cell numbers were counted. Note: **P < 0.01, ***P < 0.005.

# Supplementary Figure 2

#

#
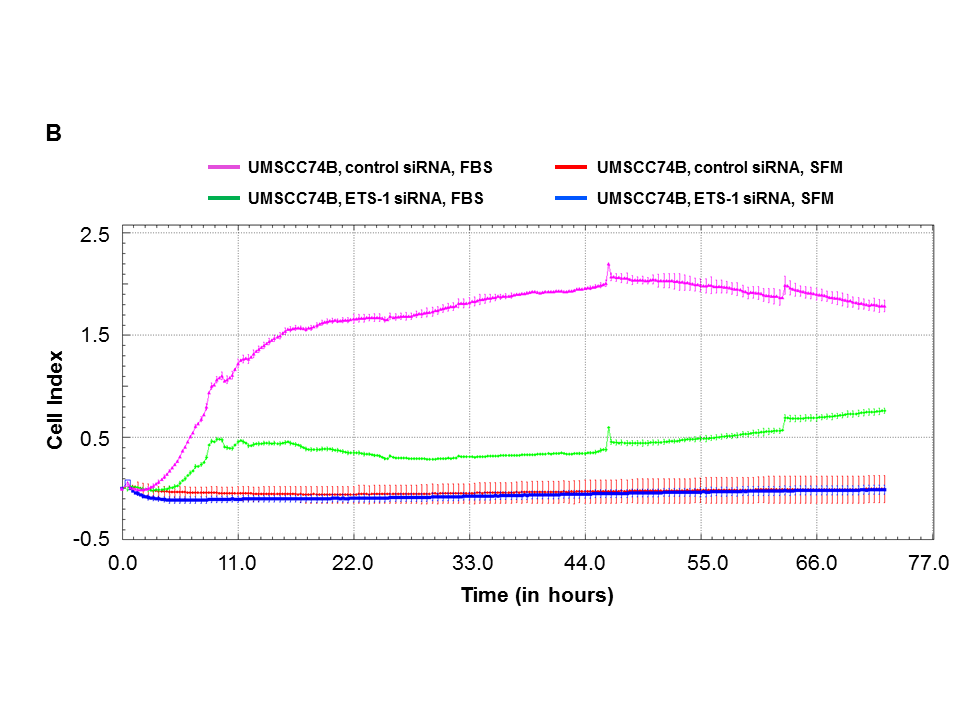

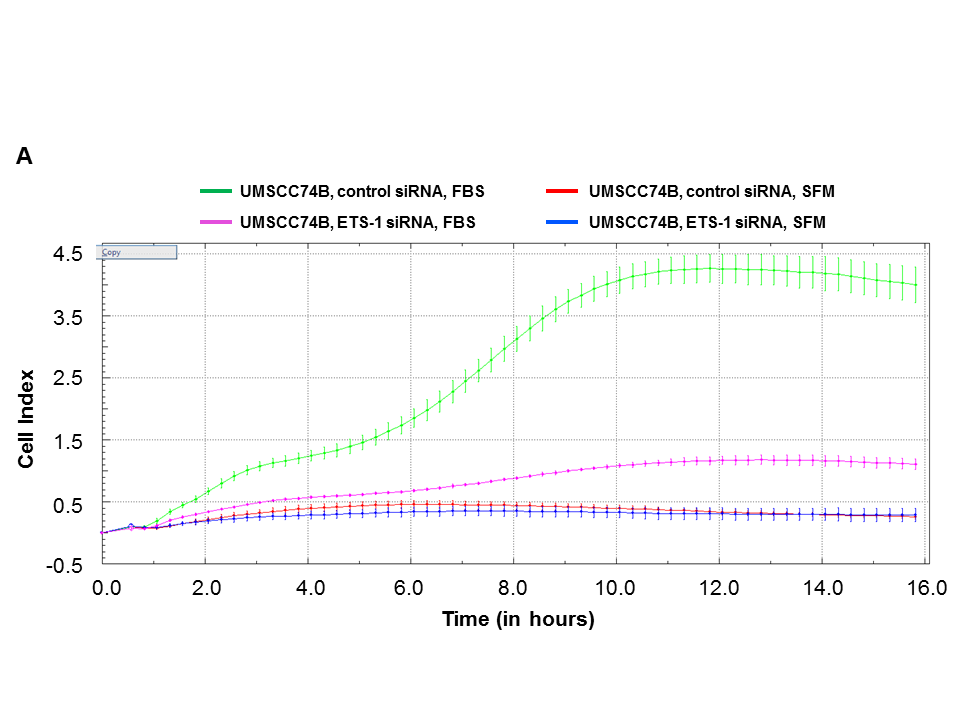


# Supplementary Figure 2 related to Figure 3

# ETS-1 is also important for UMSCC74B cell migration and invasion.

# *A*. UMSCC74B cells were transfected with non-target siRNA or siRNA against ETS-1 for 48 hours and cell migration (*A*) and invasion (*B*) were monitored by the xCELLigence real-time cell system. Note: SFM: serum-free medium

**Supplementary Figure 3**

**
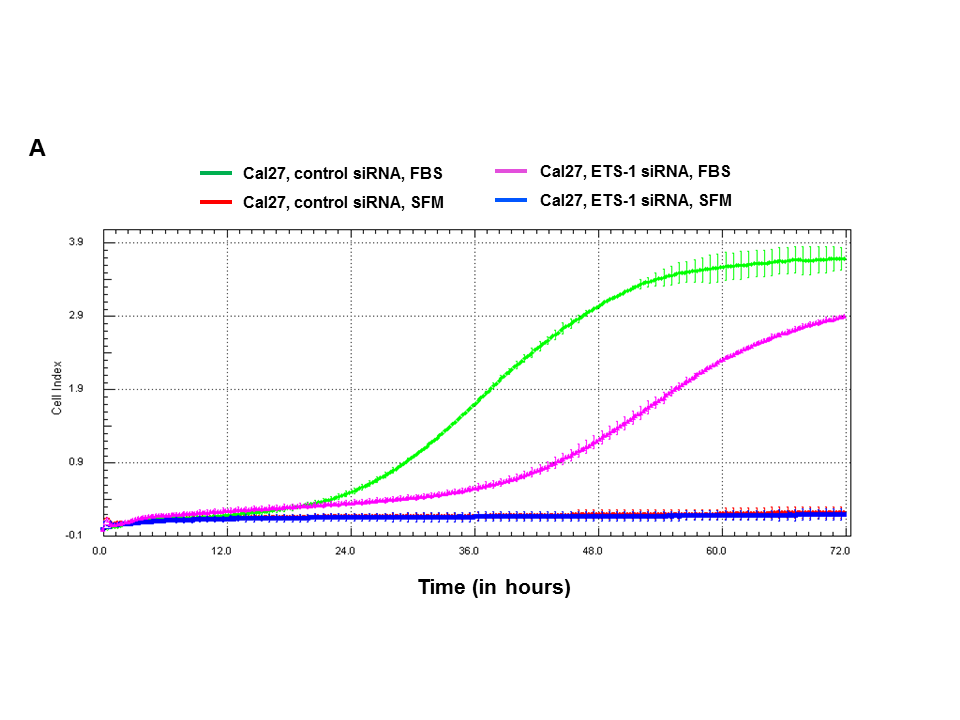
**

#
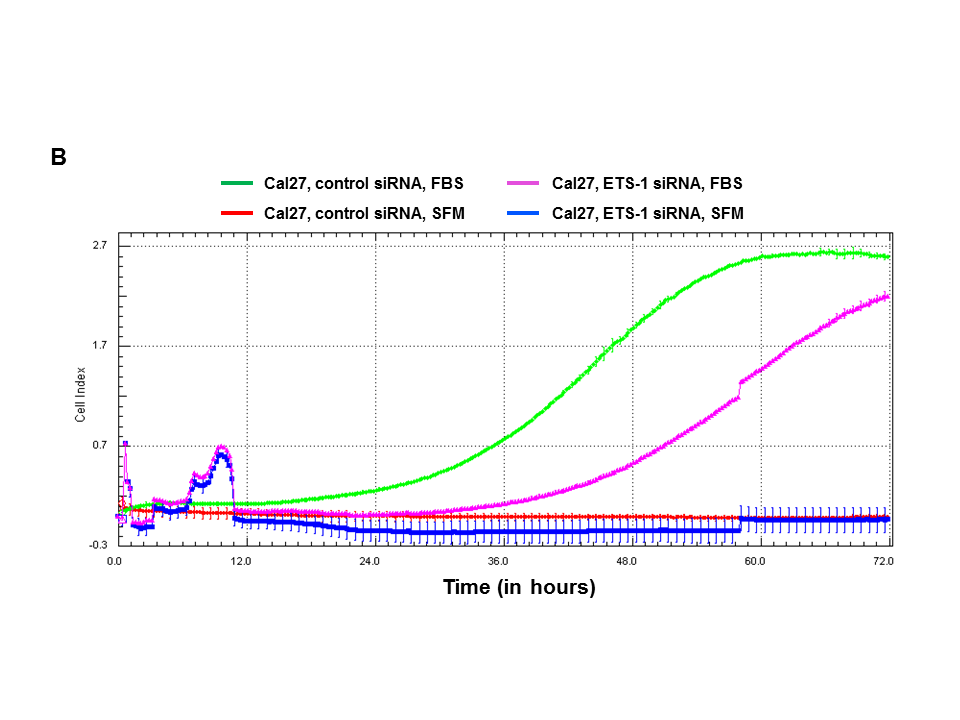


# Supplementary Figure 3 related to Figure 3

# ETS-1 is important for Cal27 cell migration and invasion.

# *A*. Cal27 cells were transfected with non-target siRNA or siRNA against ETS-1 for 48 hours and cell migration (*A*) and invasion (*B*) were monitored by the xCELLigence real-time cell system. Note: SFM: serum-free medium.

# Supplementary Figure 4

#
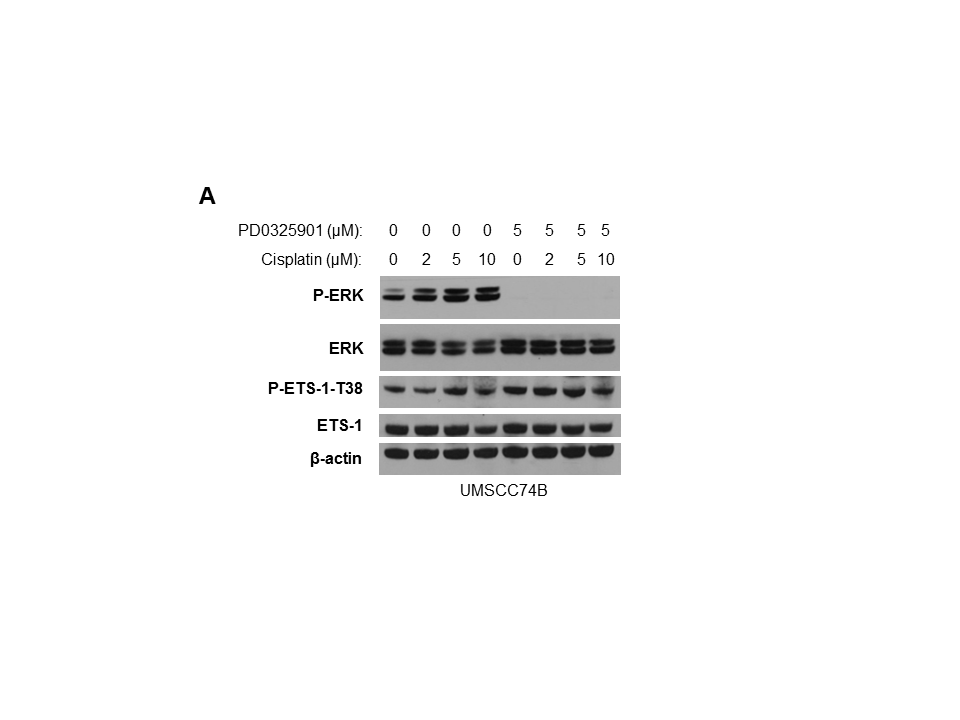


#
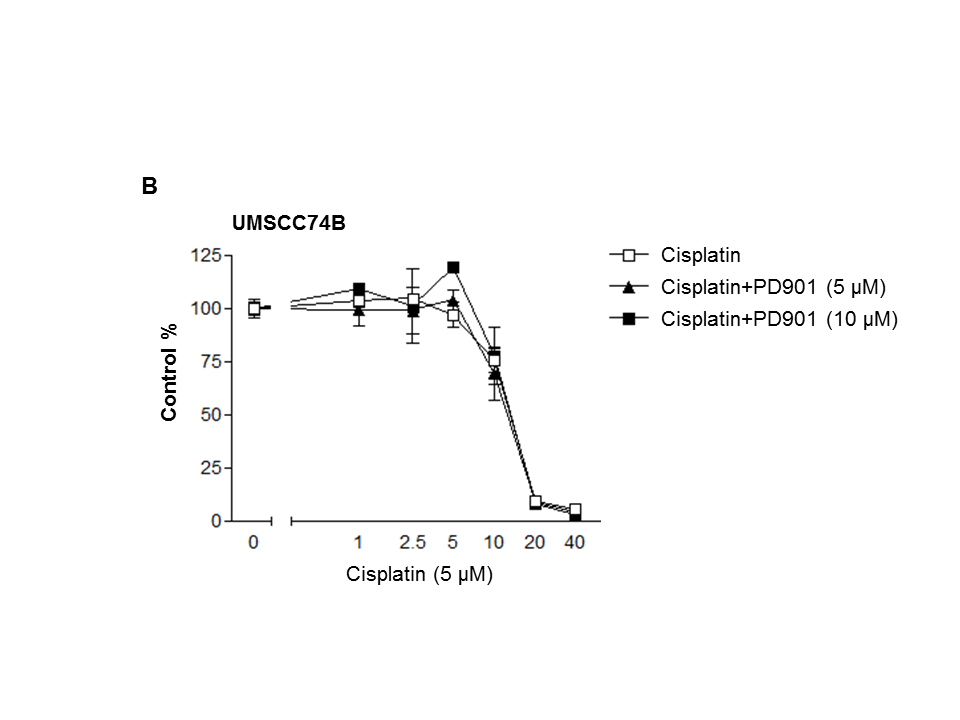


# Supplementary Figure 4 related to Figure 5

# MEK/ERK inhibitor, PD0325901, does not re-sensitize cisplatin-resistant UMSCC74B cells to cisplatin treatment.

*A*. UMSCC74B cells were treated with different doses of cisplatin alone, or in combination with PD0321901 (5 μmol/L) for 24 hours, and phosphorylation of ERK and ETS-1-T38 and total levels of ERK, ETS-1 and β-actin were detected by Western blot analysis. *B.* UMSCC74B cells were treated with DMSO, cisplatin, PD0325901, or in combination for 72 hours. Cell proliferation was measured by MTS assay.

# Supplementary Figure 5

#
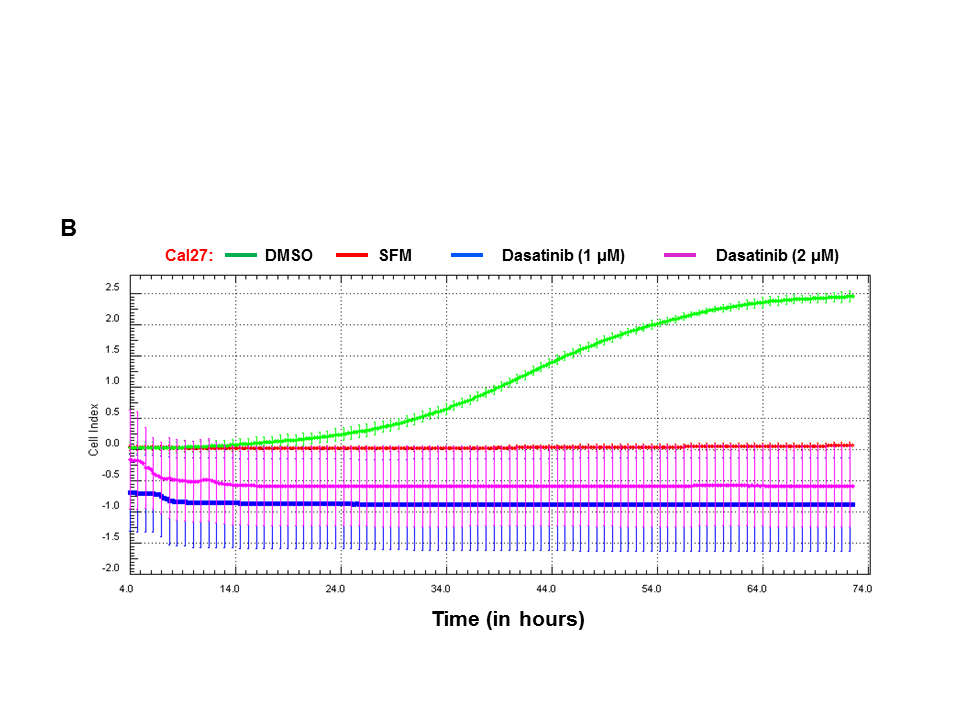

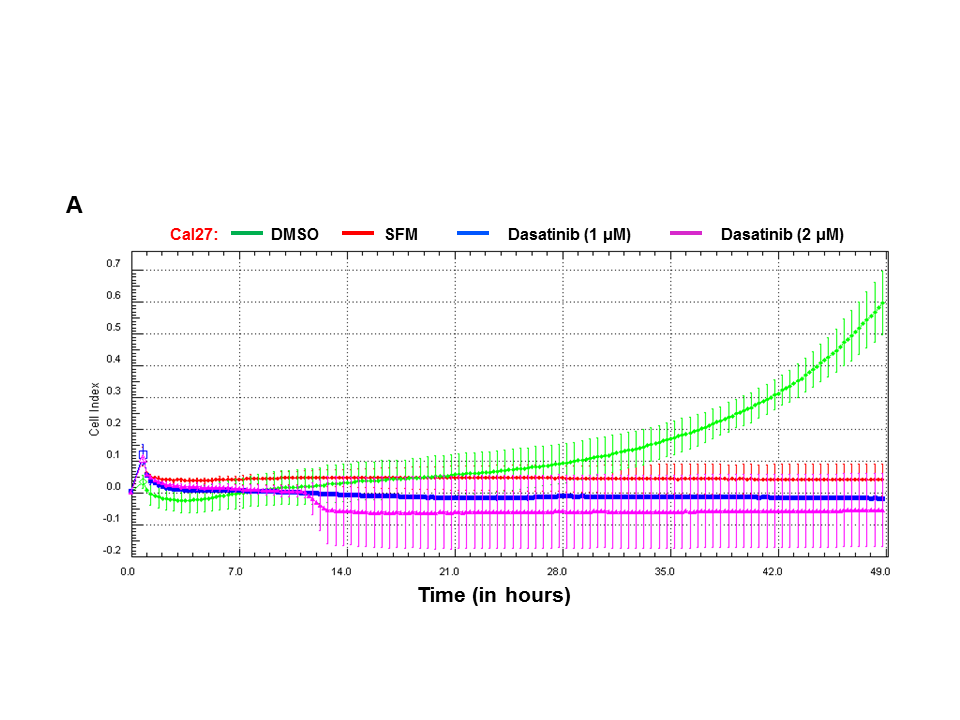


# Supplementary Figure 5 related to Figure 7

# Dasatinib inhibits cisplatin sensitive Cal27 cell migration and invasion.

*A* and *B*. Cal27 cells were treated with DMSO control or 1.0 or 2.0 μM Dasatinib and cell migration (A) and invasion (B) were monitored by the xCELLigence real time cell system. Note: SFM: serum-free medium.

# Supplementary Figure 6

#
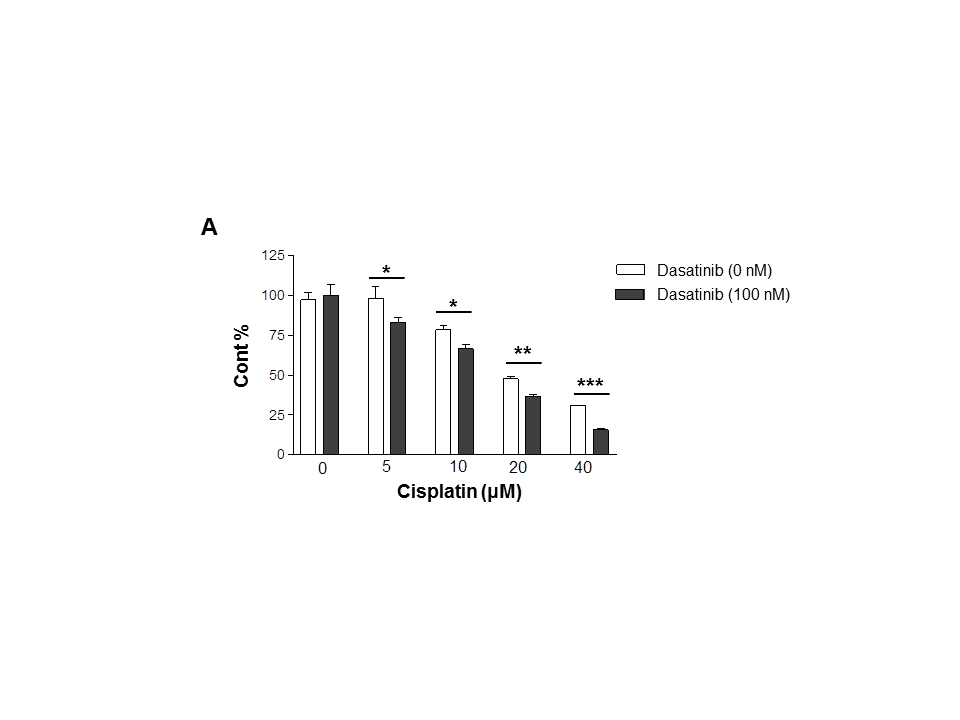


#
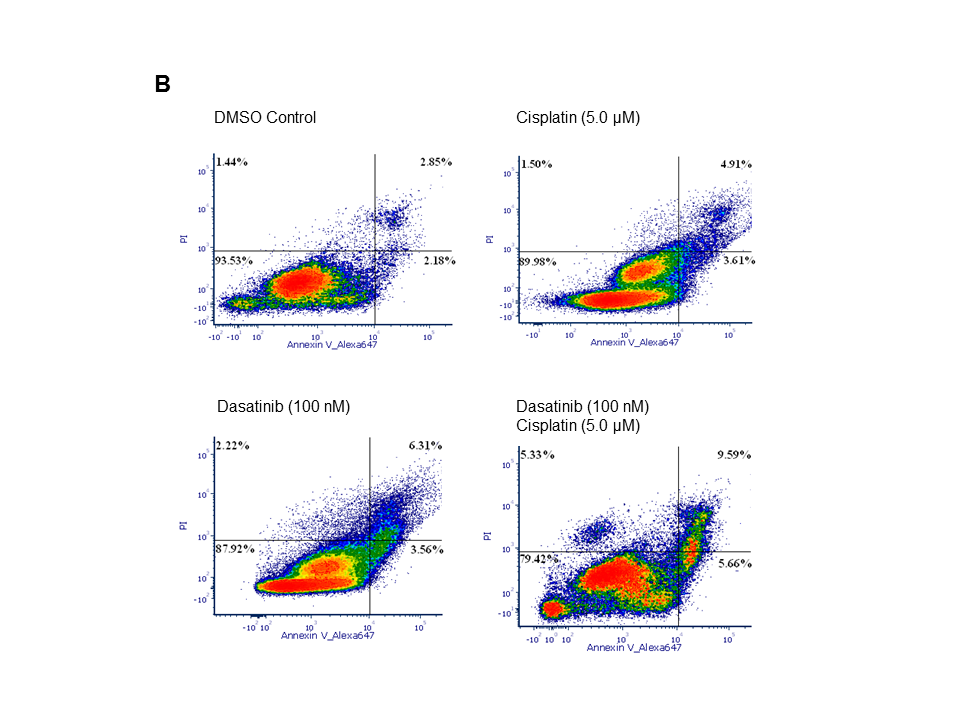


# *
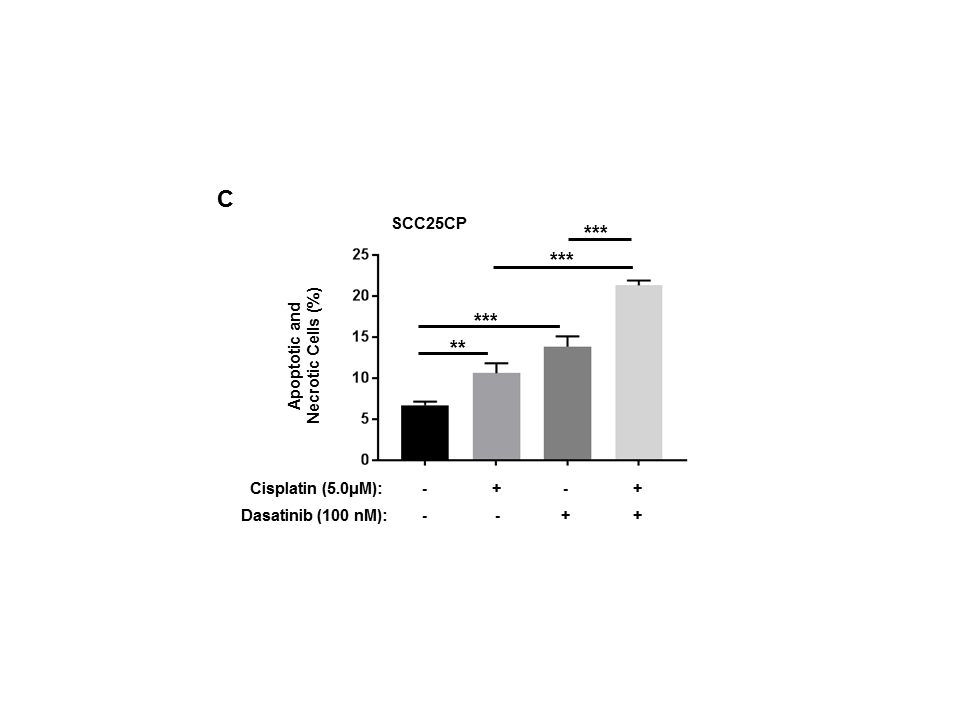
*

# Supplementary Figure 6 related to Figure 8.

# Dasatinib does not synergize with cisplatin to induce apoptosis in SCC25CP cells.

*A.* SCC25CP cells were treated with DMSO, different doses of Dasatinib, or a combination for cisplatin for 72 hours and cell proliferation were measured by MTS assay. *B.* Cells were treated with DMSO, cisplatin, Dasatinib, or a combination for 48 hours. Apoptosis was analyzed by Annexin V staining and flow cytometry. *C*. Experiments in *B* were performed in triplicate and statistical analysis was performed. P values < 0.05 were considered to be statistically significant. Note: *P < 0.05, **P < 0.01, ***P < 0.005.
